# Interconnection between Cardiac Cachexia and Heart Failure—Protective Role of Cardiac Obesity

**DOI:** 10.3390/cells11061039

**Published:** 2022-03-18

**Authors:** María Elena Soto, Israel Pérez-Torres, María Esther Rubio-Ruiz, Linaloe Manzano-Pech, Verónica Guarner-Lans

**Affiliations:** 1 Department of Immunology, Instituto Nacional de Cardiología “Ignacio Chávez”, México City 14080, Mexico; maria.soto@cardiologia.org.mx; 2 Department of Cardiovascular Biomedicine, Instituto Nacional de Cardiología “Ignacio Chávez”, México City 14080, Mexico; israel.perez@cardiologia.org.mx (I.P.-T.); loe_mana@hotmail.com (L.M.-P.); 3 Department of Physiology, Instituto Nacional de Cardiología “Ignacio Chávez”, México City 14080, Mexico; esther.rubio@cardiologia.org.mx

**Keywords:** cardiac cachexia, heart failure, adipose tissue, cardiac fat tissue

## Abstract

Cachexia may be caused by congestive heart failure, and it is then called cardiac cachexia, which leads to increased morbidity and mortality. Cardiac cachexia also worsens skeletal muscle degradation. Cardiac cachexia is the loss of edema-free muscle mass with or without affecting fat tissue. It is mainly caused by a loss of balance between protein synthesis and degradation, or it may result from intestinal malabsorption. The loss of balance in protein synthesis and degradation may be the consequence of altered endocrine mediators such as insulin, insulin-like growth factor 1, leptin, ghrelin, melanocortin, growth hormone and neuropeptide Y. In contrast to many other health problems, fat accumulation in the heart is protective in this condition. Fat in the heart can be divided into epicardial, myocardial and cardiac steatosis. In this review, we describe and discuss these topics, pointing out the interconnection between heart failure and cardiac cachexia and the protective role of cardiac obesity. We also set the basis for possible screening methods that may allow for a timely diagnosis of cardiac cachexia, since there is still no cure for this condition. Several therapeutic procedures are discussed including exercise, nutritional proposals, myostatin antibodies, ghrelin, anabolic steroids, anti-inflammatory substances, beta-adrenergic agonists, medroxyprogesterone acetate, megestrol acetate, cannabinoids, statins, thalidomide, proteasome inhibitors and pentoxifylline. However, to this date, there is no cure for cachexia.

## 1. Introduction

Cachexia is a syndrome of metabolic origin that involves involuntary and severe loss of edema-free muscle mass and that may or may not be accompanied by reduction of fat mass. It develops after chronic diseases including cancer and after other inflammatory conditions such as heart failure (HF), chronic obstructive pulmonary disease and chronic kidney disease (CKD) [1,2]. It is also present in infectious diseases including acquired immunodeficiency syndrome, sepsis [1,2] and coronavirus infectious disease 19 [3,4]. When it results from congestive HF, it is known as cardiac cachexia [5], and a vicious cycle is established between the loss of skeletal muscle mass and cardiac mass.

Cachexia and cardiac cachexia are exceptional conditions in which fat accumulation, instead of constituting a risk factor, becomes protective [6,7]. Fat accumulation in the heart is known as cardiac visceral obesity and includes epicardial adipose tissue, intramyocardial fat and cardiac steatosis [6]. It has been associated with the development of ischemic cardiomyopathy, hypertension, atherosclerosis, atrial fibrillation, cardiac microcirculatory dysfunction, and diabetic cardiomyopathy [8]; however, it reduces the risk of cardiac cachexia in HF [7]. This situation resembles the condition known as the “obesity paradox”. Although some authors have proposed that this term should be abandoned [9], it used to refer to the loss of body mass, even in overweight or obese patients, worsening the prognosis of HF [6,10]. The obesity paradox has also been related to the description of obese but metabolically healthy individuals in contrast to obese and unhealthy subjects [11]. Although cachexia is related to sarcopenia, the obesity paradox is not present in the latter condition.

Here, we analyze the mechanisms underlying cachexia and particularly cardiac cachexia and its interconnection to HF. We also discuss the protective role of cardiac obesity against this condition. We set the basis for possible screening methods, which may allow for a timely diagnosis of cachexia since there is still no cure for this condition. We also discuss the possible therapeutic measures that have been proposed for this disease

## 2. Cachexia in Heart Failure

Global cachexia, including cancer induced and cardiac cachexia, is a generalized process that involves edema-free muscle loss but may also affect the adipose tissue and other energetic reserves. It may also involve the bones [12]. According to the 2008 criteria, cardiac cachexia is characterized by a loss of 5% of body weight during the last 12 months, without edema, a body mass index of <20 kg/m^2^ and the presence of at least three of the following clinical findings: decreased muscular strength, anorexia, low body mass free of fat, fatigue, elevated reactive C protein and IL-6, hypoalbuminemia < 3.2 g/dL, hemoglobin < 12 g/dL and anemia [13]. Cachexia is a nutritional syndrome because nutrients such as protein and calories are required to reverse it, and anorectic components participate in it [12].

Since there are several definitions and methods to diagnose cachexia, the reported prevalence varies significantly. Nevertheless, the overall prevalence of cachexia may be considered to be approximately 1% of the global patient population, and the rate increases to 50–80% in cancer patients [2]. Almost 80% of cancer patients suffering cachexia will die within 1 year of diagnosis [12,14,15]. Cachexia is also related to the cardiac atrophy, which is commonly seen in cancer patients. Cardiac cachexia is a complex, severe multifactorial condition which has been linked to chronic HF [6], and it is an important predictor of poor clinical prognosis, reduced survival, and elevated hospital costs [14,15,16].

HF is a progressive and chronic ailment that disrupts the pumping of the heart or its filling. The main cause of HF is coronary artery disease. It has been divided into three subtypes, which are HF with reduced ejection fraction, HF with midrange ejection fraction and HF with preserved ejection fraction. This classification also considers natriuretic peptide levels and the presence of structural heart disease and diastolic dysfunction. Symptoms may range from mild to severe and include difficulty in breathing, fatigue, swelling of the legs and accelerated cardiac rhythm [13]. Congestive HF specifically refers to the most severe stage in which fluid accumulates within the heart and results in poor pumping capacity. Congestive HF affects subjects of all ages, from children to the elderly. Almost 1.4 million patients with chronic HF are under 60 years of age, and congestive HF is present in 2 percent of persons aged 40 to 59. Patients have a five-year survival rate of about 50% for all stages. Comorbidities of HF have an important influence on the clinical course of the disease and render patients more vulnerable to nutritional deficiencies. These comorbidities include coronary artery disease, hypertension, chronic obstructive pulmonary disease, chronic renal disease, diabetes mellitus and anemia, among others where the ejection fraction is conserved [15]. HF is a curable condition, and treatments include changes in nutrition reducing salt intake, limiting fluids and prescription drugs. In some cases, the placement of a defibrillator or a pacemaker are needed [13].

**Figure 1:**
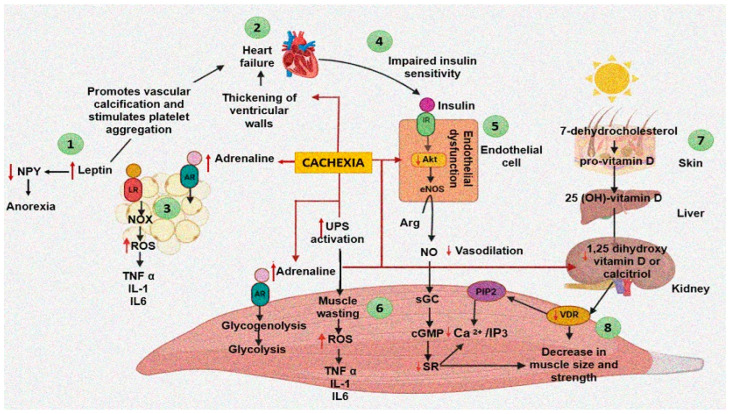
Events and processes generated by cardiac cachexia and heart failure and interrelation between different organs (skeletal muscle, heart, adipose tissue, endothelial cells, kidney, and liver) and their influence on the appearance of cachexia. (**1**) Leptin promotes a prothrombotic state. (**2**) Heart failure leads to alterations in the synthesis and degradation of cardiac proteins. (**3**) Leptin promotes an inflammatory environment. (**4**) Impaired insulin sensitivity as a result of heart failure. (**5**) The decrease in insulin sensitivity as a consequence of cachexia favors endothelial dysfunction. (**6**) Muscle wasting as a result of increased UPS and an inflammatory environment due to cachexia. (**7**,**8**) Vitamin D and VDR deficiency affect muscle structure. Abbreviations: Arg = Arginine, AR = Adrenaline receptor, cGMP = Cyclic guanosine monophosphate, eNOS = Endothelial nitric oxide synthase, IL = Interleukin, IR = Insulin receptor, LR = Leptin receptor, NO = Nitric oxide, NOX = Nicotinamide adenine dinucleotide phosphate oxidase, NPY = Neuropeptide Y, PIP2 = Phosphatidylinositol biphosphate, IP3 = Inositol triphosphate, ROS = Reactive oxygen species, SR = Sarcoplasmic reticulum, TNFα = Tumor necrosis factor alpha, UPS = Ubiquitin–protease system, VDR = Vitamin D receptor.

Cardiac cachexia may appear independently from age, ventricular function, or functional classification of HF. Wasting of the cardiac and skeletal muscles is linked to metabolic alterations. Decreased cardiac muscle mass may result from skeletal muscle cachexia. The loss of cardiac muscle leads to a poor pumping capacity, and therefore, supply of oxygen and nutrients to the rest of the body is decreased. In turn, the loss of skeletal muscle mass and the presence of edema complicate the return of blood to the heart and cardiac filling and, therefore, the pumping capacity of the heart. Thus, a vicious cycle is established. The presence of cardiac cachexia is related to elevated morbidity and mortality associated to weight loss and systemic inflammation [6]. The interrelationship between cachexia, HF and obesity is complex, and the effect of cachexia on different organs and tissues is illustrated in Figure 1 and discussed in the next paragraphs.

### Conditions Associated with Heart Failure and Cardiac Cachexia

Several conditions are associated with HF and cardiac cachexia which include fatigue, dyspnea, anorexia and anemia which are discussed in this section.

Fatigue is one of the most frequent symptoms of HF. It is related to exhaustion, lack of energy and limitations to maintain and autonomous and independent lifestyle [17,18]. The physiopathological causes of fatigue in HF are multifactorial including a low cardiac output, poor tissue perfusion, dysfunction of the autonomic nervous system, poor physical condition, and endothelial dysfunction.

Dyspnea is present in HF and significantly increases energy consumption being a cause of anorexia and swallowing disorders. In patients with restricted diets, polymedication contributes to these alterations. Furthermore, intestinal edema and hepatic and intestinal congestion may cause early satiety, nausea and therefore, anorexia and intestinal malabsorption of nutrients [19].

Anorexia is a symptom present in 72% of cases of cachexia and is related to HF through fatigue and dyspnea; nevertheless, it is a symptom which should be analyzed with caution since it is also related to the use of drugs, depression, and gastrointestinal problems [20,21].

Anemia is a common condition in patients with HF. It causes a reduction in oxygen supply to the periphery, which contributes to exercise intolerance, and is associated with greater clinical severity, HF, rapid deterioration in the evolution of patients and increased mortality. In patients with cachexia, the prevalence is between 24% and 70%, regardless of the functional class [22].

Therefore, fatigue, dyspnea, anorexia, and anemia are the most common conditions associated with HF and cardiac cachexia.

## 3. Obesity, Cardiac Obesity and Cardiac Cachexia

Cardiac cachexia is a condition which is diminished in the presence of cardiac obesity. Therefore, in this section we discuss the concepts of obesity and cardiac obesity and their interconnection with cardiac cachexia. Obesity and overweight are defined as excessive fat accumulations that constitute risk factors for the development of many diseases. A body mass index (BMI) of over 25 is considered as overweight, while a value over 30 is considered as obesity. Fat tissue may accumulate subcutaneously or in the viscera. Normally, subcutaneous adipose tissue (SAT) constitutes 85% of total adipose tissue mass in obese and lean individuals, while visceral adipose tissue (VAT) represents 15%. However, regarding fat tissue, quantity is less important than quality, since there are metabolically healthy but obese individuals who resist the negative impact linked to excessive subcutaneous body fat and metabolically obese but normal weight subjects who have abnormal metabolic manifestations related to increased levels of visceral fat and low levels of subcutaneous fat [23].

There is an association between obesity, metabolic syndrome, cardiomyopathy, and HF. Cardiac adipocytes are metabolically active and secrete proinflammatory cytokines having autocrine and paracrine functions. They also lead to altered bioavailability of adipokines, which result in adipocyte hypertrophy, tissue hypoxia, inflammation, and oxidative stress [24,25,26]. Obesity induces inflammatory processes and results in obesity-related syndromes that include diabetes mellitus, hypertensive heart disease, coronary artery disease, ischemic cardiomyopathy, diabetic or metabolic or obesity-related cardiomyopathy, coronary microcirculatory dysfunction, and atrial fibrillation [27]. When myocardial dysfunction develops in obese individuals in the absence of HF, patients are diagnosed as having obesity cardiomyopathy [28]. Nevertheless, in the presence of HF, obesity is implied in 11% of cases in men and 14% in women [29]. Obesity may directly participate in HF by causing structural and functional changes in the heart that lead to cardiac dysfunction and hemodynamic and myocardial changes. It may also participate indirectly in HF through an increased susceptibility to other risk factors. Elevated fat tissue in the heart seems to protect against cardiac cachexia [6,7].

Low amounts of adipose tissue deposits in the heart are also associated to heart diseases [30]. When cardiac adipose tissue is scarce, the heart morphology is altered as in chronic metabolic disorders and in some hereditary conditions such as generalized congenic lipodystrophy. In a postmortem study on 85 cases with atrophic hearts from different pathologies, total mass was decreased in 52% of the hearts, and the subepicardic adipose tissue was reduced or absent. In 14% of the atrophied hearts, there was fat infiltration, cells were small and partially collapsed and there were vacuolated spaces in the peripheric spaces. Cells had hyperchromatic nucleuses [31]. Autopsies from patients with generalized lipodystrophy had normal or slightly hypertrophic myocytes, but there was reduced perivascular adipose tissue [32], and lipid content in the myocardium was increased threefold [33].

Cardiac visceral adipose tissue, which is a form of visceral adipose tissue, is composed of local visceral epicardial adipose tissue, intramyocardial visceral fat tissue and cardiac steatosis [6]. Epicardial adipose tissue constitutes about 20% of cardiac weight and surrounds the heart. It is in contact with the epicardial conductive coronary artery. Epicardial adipose tissue has an elevated rate of synthesis of free fatty acids and of their incorporation and degradation depending on myocardial needs [19]. It provides many adipokines with proinflammatory and proatherogenic effects [34,35]. In obesity, it becomes hypoxic and dysfunctional, losing its capability to store triglycerides and decreasing lipolysis [19,36]. Epicardial adipose tissue also facilitates coronary artery calcification [37].

Intramyocardial visceral fat tissue is found in the right ventricle, with a very small presence in the apical part of the left ventricle. Its endocrinological role in heart disease and its relation to conductive system disturbances remains unknown; however, it plays a role in the electrophysiological remodeling of the ventricle, and it is involved in endocardial scarring [38,39].

The abundance of cardiac steatosis depends on the presence of obesity and type-2 diabetes mellitus, and it increases with age. This type of cardiac fat is an endogenous source of cytosolic free fatty acids. Circulating free fatty acids participate in the regulation of intramyocardial fat depots [40,41]. Excessive uptake of fatty acids activates their oxidation and elicits lipotoxicity, causing impaired cardiac function [42,43]. The presence of cardiac obesity reduces the risk of cardiac cachexia [6,7].

## 4. Cardiac Cachexia, Heart Failure, Frailty and Aging

Cachexia and cardiac cachexia are often associated to other conditions present during aging such as frailty, loss of appetite and social factors such as depression and dementia. Aging is also related to changes in hormone levels such as testosterone and cortisol which contribute to cachexia. These topics are discussed in the present section.

Frailty is becoming a global health problem impacting clinical practice. Its prevalence is increasing alongside aging in the population. In turn, the occurrence of HF is also increasing in the elderly, although it can be present at any age [44]. Moreover, the progression to severity of the HF is common in patients with sarcopenia, cachexia, and frailty [45]. Frail patients with HF are often predisposed to poor outcomes, and therefore, frailty is a relevant prognostic factor.

Frailty is characterized by a diminution in the functioning of many physiological systems and an elevated vulnerability to stressors. There is a progressive decline in cognitive function, loss of appetite and low protein intake [46]. The risk of developing frailty increases in patients with comorbidities, poor diet, sedentary lifestyles, and low socioeconomic position [47]. There is also a reciprocal interaction between depression and frailty in older adults [48].

Subjects with frailty have elevated risk of mobility limitation, falls, fractures, increased length of hospital stay, hospital readmission and mortality [47]. Frailty overlaps with sarcopenia and cachexia, and the risk of these syndromes is particularly high due to acute illness including HF, immobility, and anorexia [49]. However, there is still no standard instrument to identify frailty [47].

Age is another predisposing factor for the development of cachexia, and this condition is multifactorial including a combination of physiological changes such as a decline in smell and taste, a reduced desire to eat, delayed gastric emptying and pathological conditions among which are depression, dementia, somatic diseases, medications and iatrogenic interventions, oral health status, dehydration, and social factors such as the poverty and loneliness [50]. Loneliness, poverty, and social isolation are the predominant social factors that contribute to decreased food intake in the elderly population. These factors may result in chronic depression, which is a common psychological problem in the elderly and a significant cause of loss of appetite [51].

Aging is also associated with a loss of sex hormone in both men (andropause) and women (menopause). In men, andropause may trigger a decline in bone mass and density and in muscle mass and strength. In women, menopause, and the decline of 17β-estradiol result in severe decalcification of bone and arthritis and contribute to activation of the inflammatory cytokines, hip fracture, pressure ulcers and a decrease of the immune function [52]. Cachectic males have greater deficits in handgrip strength when compared to cachectic females [53].

Cortisol plays an important role in the development of depression, which is a complex, multifactorial and heterogenous disease with a high world prevalence, and the levels of this hormone are increased during aging. This hormone impacts cognitive capacity and attention [54]. Furthermore, an elevation in the seric concentration of cortisol favors the inflammatory response and the presence of oxidative stress, which, in turn, participate in the development of pathologies such as HF and cachexia [55].

Cachexia and homeostatic disturbances are associated with CKD [56], persistent chronic inflammation and hypoalbuminemia [57,58]. Elevated circulating levels of cytokines, such as leptin, are an important cause of uremia-associated cachexia via signaling through the central melanocortin system [59].

Cardiac cachexia overlaps with sarcopenia, frailty, and cardiac atrophy, which are conditions commonly found in the elderly population. Changes in hormones including sexual hormones and others may participate in the development of these conditions, and they, in turn, affect other functions in the body associated with mental disturbances and CKD.

## 5. Pathogenesis of Cachexia

### 5.1. Cachexia and the Balance between Protein Synthesis and Breakdown in Heart Failure

The molecular pathways involved in the development of cachexia in skeletal muscle and in the heart depend on different metabolic conditions and physiopathological factors. Muscle and cardiac mass depend on fiber protein content, which is regulated by the balance between protein breakdown and synthesis [60]. Cardiac hypertrophy appears before the onset of HF and represents a response of the heart to an elevated workload. Cardiac hypertrophy is characterized by an increase in the size of cardiomyocytes and thickening of ventricular walls. In the beginning, this growth is an adaptive response to maintain cardiac function; nevertheless, with time, these changes become maladaptive and cause HF, in which the muscle mass of the heart is significantly decreased, the walls of the ventricles become thin, and the pumping capacity of the heart is limited [61].

Although the function of each cardiomyocyte is maintained throughout life, its proteins and organelles are constantly renewed. Every heart protein is regenerated approximately every 30 days, and amino acids are employed in the synthesis of new protein [62]. Proteins in the heart include those with structural, nuclear, and enzymatic function and the contractile proteins, which are responsible for the main function of the heart [63]. Therefore, turnover includes actin and myosin, and it is employed to eliminate proteins with normal “wear and tear” and to remove misfolded proteins [62].

Protein synthesis in skeletal and cardiac muscle is determined by dietary protein intake and by the regulation of anabolic molecules. Although a decrease in muscle and cardiac protein synthesis after protein ingestion during aging is still controversial [64], there is a decrease in insulin sensitivity [65]. The impaired insulin sensitivity during cachexia, which is similar to that present in aging, may be due to endothelial dysfunction and a lower expression of the Akt and mTOR pathways, which form part of the downstream intracellular signaling of insulin (Figure 1). Both sarcopenia and cachexia are also associated with lower levels of the insulin-like growth factor 1 [12]. The mechanisms involved in protein synthesis are elevated with afterload; however, preload stress does not have the same effect. During preload, myofibrils lengthen, while in afterload, they thicken [63].

Proteins are degraded through autophagy, which is the breakdown of components of the cytosol such as organelles and proteins within lysosomes and vacuoles. Protease systems, such as the ubiquitin–protease system (UPS), the lysosomal autophagy pathway and the calpain system, participate in the degradation of proteins and organelles. A significant role in contractile dysfunction and programmed cell death is played by the imbalance in degradation of ubiquitinated protein conjugates in failing cardiomyocytes [12]. An increased activation of the UPS seems to play the most important role for inducing muscle wasting in cachectic conditions (Figure 1). Another mechanism that activates UPS-mediated protein degradation is a downregulation of deubiquitinases to promote ubiquitination [66,67].

The pathological activation of the protease calpain in cardiomyocytes results in the degradation of the cytoskeleton, affecting contraction [64]. Calpains form part of a large family of calcium-dependent cysteine proteases that may cut myofibrillar proteins, altering the sarcomere, and they are regulated by their inhibitor calpastatin [68,69]. The calpain system participates in the worsening HF through a different mechanism that includes the degradation of titin [64]. Titin forms part of the contractile apparatus and contributes to the intracellular stiffness of the myocytes [62]. An alteration in the regulation of the calpain family of proteases has been tested to improve HF outcomes.

Increased inflammation and a high level of reactive oxygen species (ROS) increase the signaling of protein degradation via numerous key pathways such as the FoxO transcription factors and induce muscle wasting. Mitochondria are the most abundant organelles in the heart, and their activity is essential to maintain muscular energetic homeostasis. Under normal conditions, there is a balance between the processes of fusion, fission and mitophagia, which regulate the amount of these organelles and favor the function of skeletal and cardiac muscle. Nevertheless, factors such as inflammation, oxidative stress and hyperglycemia alter the mitochondrial dynamics and are associated with cachexia [70,71,72,73].

### 5.2. Role of the Endocrine System in Heart Cachexia

Neurohormones have been included among the mechanisms that regulate the synthesis and degradation of muscle fibers since alterations in their concentrations may contribute to an imbalance in the anabolic and catabolic pathways [74]. Hormonal signals involved in cachexia include insulin and insulin growth factor 1 (IGF-1), leptin, ghrelin, melanocortins, neuropeptide Y and growth hormone [12]. Anabolic agents involved are impairments in the growth hormone/insulin-like growth factor-I axis and insulin resistance (Figure 1). Catabolic agents that participate include increased levels of catecholamines and an increased cortisol/dehydroepiandrosterone ratio [75].

Insulin is one of the most important anabolic regulators being stimulated by muscle hypertrophy, which induces the secretion of insulin growth factor 1 (IGF-1) and the triggering of the PI3K–Akt–mTOR pathway. IGF-1 is an anabolic growth factor that elevates the proliferation of satellite cells and the synthesis of protein [76,77]. It also abolishes protein degradation [78,79,80]. IGF-1 acts by inhibiting the protein synthesis by decreasing the PI3K–Akt–mTOR pathway. This pathway is inhibited by myostatin by linking to its receptor activin A receptor type B to further stimulate gene transcription via “small Mothers Against Decapentaplegic” (SMAD2 or SMAD), which are transcriptional factors related to similar proteins in *Caenorhabditis elegans*, and the signaling of peroxisome proliferator-activated receptor-gamma coactivator 1 alpha (PGC-1α), which is a key regulator of mitochondrial biogenesis [81]. Cachectic HF patients have raised plasma levels of norepinephrine, epinephrine, and cortisol, and they show high plasma renin activity and increased plasma aldosterone levels [82,83].

During cachexia, there is also an inappropriate hypothalamic response to the mechanisms controlling energy homeostasis. Leptin and ghrelin have many different effects on the cardiovascular system. Leptin, at the level of the central nervous system, increases the activity of the sympathetic nervous system, causing a pressor effect. However, peripherally, it induces vasodilation by an endothelium-dependent mechanism. Ghrelin, in contrast, decreases sympathetic activity, having a depressor effect, and at the vessel level, ghrelin produces vasodilation. Ghrelin improves left ventricular function and cardiac cachexia in HF [84] (Figure 1).

Leptin impacts cardiac cachexia and obesity-related cardiomyopathy by many mechanisms. Leptin is proinflammatory and proliferative, and it promotes calcification in the blood vessels; it may also stimulate platelet aggregation and inhibit coagulation and fibrinolysis (Figure 1). In contrast, ghrelin has anti-inflammatory effects in the vasculature [85]. Despite the changes in the leptin and ghrelin concentrations, energy intake is not increased due to a persistent activation of the proopiomelanocortin (POMC) system, which is anorexigenic. A decreased activity of the neuropeptide Y (NPY, orexigenic) neurons may also contribute to the maintenance of energy intake [76].

### 5.3. Role of Micronutrients and Vitamin D in Cachexia

The degree of progression of cachexia is strongly linked to deficiency in the concentration of Vitamin D. Moreover, micronutrient deficiencies, including deficiency of Vitamin D are potential contributing factors to the progression of HF, and unintentional cachexia is associated with worse survival in HF patients [86] (Figure 1). Vitamin D is mainly obtained through cutaneous synthesis, during exposure to ultraviolet rays (70–80%), and the rest is acquired through by the ingestion of food including sardines and milk, among others. Skin ultraviolet exposure induces the conversion of 7-dehydrocholesterol to pro-Vitamin D, which is synthesized by the liver from cholesterol. It is subsequently transported to the liver, where it is hydroxylated to 25(OH)-Vitamin D and is then released to the circulation, reaching the kidney, where it is again hydroxylated by the 1-alpha-hydroxylase to convert it to 1,25 dihydroxy Vitamin D or calcitriol, which is the active form of Vitamin D. This vitamin has autocrine and paracrine actions on different organs and systems such as intestines, muscular tissue, heart, lungs, parathyroid glands, and kidneys, among others [87]. Patients with cachexia show deficiency in intestinal absorption associated with low levels of the Vitamin D. This is due to a deficiency in the intestine proton pump inhibitors that contribute to the reduction of muscle mass. The deficiency of proton pump inhibitors is associated to long-term the use of nonsteroidal anti-inflammatory drugs, which decrease the absorption of several mineral tracers such as magnesium. Magnesium is necessary for activation of Vitamin D. Low Vitamin D and magnesium levels can lead to the increased inflammation involved in muscle wasting [88].

Given the lipidic nature of calcitriol, it enters the cells by diffusion through the membrane, and it then joins to a nuclear transcription factor known as the Vitamin D receptor (VDR). The VDR receptor mediates both the nongenomic and genomic effects of Vitamin D [89]. To generate the genomic effects, the VDR joins to another nuclear receptor, the retinoic acid X, and this complex binds to small DNA sequences, initiating cascades of molecular interactions that modulate the transcription of specific genes that regulate calcium metabolism, phosphate intestinal absorption and bone mineralization.

The deficiency of VDR may lead to hypertrophy, loss of muscle mass and a decrease in the contractility of myocytes [90]. The VDR expression varies in muscle tissue depending on age, sex, and the presence of pathological conditions [90]. In addition, both the Vitamin D deficiency and low level of VDR are associated with important effects on muscle size and strength and, therefore, to muscular weakness [91]. Mice with deletion of the Vitamin D receptor in myocytes have sarcopenia and impaired muscle function [92]. Vitamin D-deficient patients show a decreased fiber diameter and area of type II fibers, and treatment with 1-α-hydroxy Vitamin D3 and calcium restores this condition. The histological analysis of skeletal muscle biopsies from adults with Vitamin D deficiency reveal enlarged interfibrillar spaces, infiltration of fat, presence of glycogen granules, fibrosis, and atrophy of type II muscle fibers [90]. In VDR-null mutant mice, there is a progressive decrease in their muscle fiber diameters [93]. Furthermore, VDR contributes to the regulation of glucose homeostasis and insulin sensitivity by modulating the expression of genes involved in metabolic syndrome and diabetes [94].

In contrast, several studies have demonstrated that patients with cachectic cancer frequently show decreased circulating Vitamin D and DVR [95], and this decrease can be associated also with poor cancer prognosis and disease progression [96].

During cachexia, there are deficiencies in micronutrients and trace elements such as calcium, magnesium, zinc, iron, thiamine, Vitamins E and K and folate [97]. Other vitamins such as Vitamin E in combination with Omega-3 fatty acids improve survival, and Vitamin C supplementation leads to an improvement of various quality of life aspects [98]. HF syndrome has been associated to selective deficiency of selenium, calcium, and thiamine. Other nutrients, particularly Vitamins C and E and beta-carotene, are antioxidants and may have a protective effect on the vasculature [99].

### 5.4. Inflammation in Cachexia

During cachexia, there is also activation of proinflammatory cytokines such as tumor necrosis factor-alpha (TNF-α), interleukin-6 and interleukin-1, which may promote the wasting process [75]. Injecting TNF-α into mice activates the ubiquitin proteasome system and impairs muscle function. Moreover, serum levels of inflammatory cytokines such as IL-6 or TNF-α are elevated and associated with muscle wasting in various diseases (Figure 1).

Moreover, obesity is now recognized as a chronic and systemic inflammatory disease. Adipose tissue is an important endocrine organ that releases a variety of factors which include cytokines (TNF-α, IL-6, IL-1β, IL-10, among many others) and adipokines which promote important systemic inflammation and are possibly associated with the pathogenesis of cachexia and cardiac cachexia [82,100]. Even though interleukins may participate in the control of muscular mass and adipose tissue, there is still information lacking on their role in cachexia [101] (Figure 1). In cardiac cachexia and HF, there is an increase in TNF-α as a result of the inflammatory state, which results in a low function of the bone marrow, insensitivity to induced production of erythropoietin and poor iron liberation [102].

### 5.5. Intestinal Malabsorption in Cachexia

**Figure 2:**
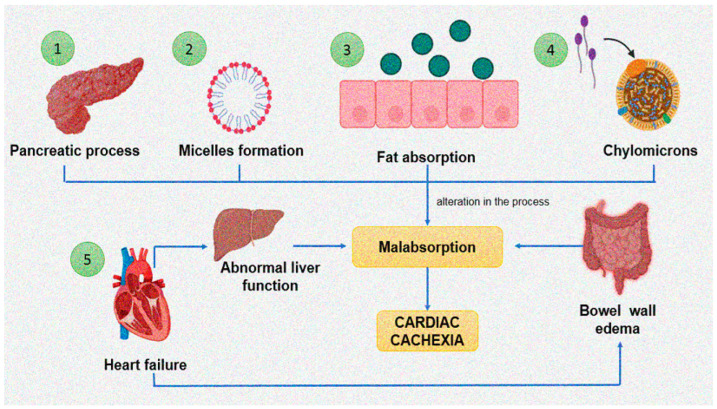
Cardiac cachexia, malabsorption syndrome and factors that favor them. (**1**) Hydrolysis of dietary triglyceride by pancreatic lipase. (**2**) The resulting fatty acids and monoglycerides then combine with bile salts from the liver to form micelles. (**3**) Dietary fats are absorbed across the mucous membrane into the intestinal cell. (**4**) The triglycerides are resynthesized and then incorporated into chylomicrons and transferred to the lymphatic system. (**5**) Heart failure generates alterations in hepatic functioning and bowel wall edema that favor malabsorption syndrome and, consequently, cardiac cachexia.

Malabsorption in the gut is associated to cardiac cachexia, and it results from bowel wall edema and a decrease in the perfusion of the bowel. It has an important impact on the development of cachexia [103]. Fat absorption is a complex process [104] that begins with the hydrolysis of dietary triglyceride by pancreatic lipase. The fatty acids and monoglycerides that are produced combine with bile salts that are synthesized in the liver to form micelles. Dietary fats are then absorbed across the mucous membrane into the intestinal cell where triglycerides are resynthesized. They are then incorporated into chylomicrons and transferred to the lymphatic system. An alteration at any of these steps results in malabsorption of fat (Figure 2). Patients with chronic HF often have abnormal liver function [105,106]. This could theoretically result in decreased production of bile salts and, consequently, fat malabsorption [107].

Colonization of the small bowel by bacteria may also be the origin of fat malabsorption [108]. Nevertheless, overgrowth of bacteria in the gut has not been found in cardiac cachexia [109].

In contrast, amino acid or protein uptake is not altered or even slightly elevated in cachexia [107,109].

Mesenteric ischemia and altered microcirculation of the gut are the cause of malabsorption in cachexia due to HF [110]. In addition to malabsorption, cachexia activates energy dissipation in futile cycles, which are initiated by an alteration in the amino acid composition that is the result of the acute phase response in the liver. Futile cycles are substrate cycles that do not have an anabolic or catabolic function but dissipate energy. They alter the liver and occur in the glycolysis/gluconeogenesis pathways [111]. Energy wasting in the liver in cachexia leads to a decrease in the capacity of mitochondria to perform oxidative phosphorylation as a result of an increase in mitochondrial cardiolipin content [112]. This is accompanied by an increase of TNF-α.

## 6. Timely Diagnosis of HF and Cachexia

The timely diagnosis for these diseases is a goal at present since there is no cure for this condition. Different detection tools have been employed in patients with HF and cachexia; however, none of them has been postulated as a gold standard [103,104,105,106,107,108,109,110,111,112,113,114,115,116,117].

The anthropometric evaluation of the body composition in patients with HF is essential due to the strong association between cachexia and poor clinical results. However, the evaluation of malnutrition in patients with HF is complicated since the loss of muscular mass and of weight may be masked by edema. Therefore, even if weight and body mass index may remain constant in routine clinical evaluations, malnutrition may exist in the frame of congestion. In patients with HF and reduced or conserved left ventricular systolic function, a low body mass index without edema is associated to an increased mortality [118]. In critically ill patients with cardiogenic shock, the American Society for Parenteral and Enteral Nutrition (ASPEN) recommends the use of the nutritional risk score and the score of critical patients (NUTRIC) in addition to the Nutritional Risk Screening 2002 (NRS 2002) to evaluate their nutritional status [119]. This is in contrast to the recommendation from the European Society of clinical nutrition and metabolism (ESPEN) [120]. The most recent consensus scheme of the Global Leadership Initiative on Malnutrition (GLIM) has also been proposed on a global scale for the diagnosis of malnutrition, which can be predictive for the renutrition syndrome [121,122]. Nevertheless, none of these tools has been specifically validated patients with HF at risk or diagnosed with malnutrition.

It has become important to detect muscle mass and to find therapies that significantly improve it in patients with HF since muscle mass predicts outcomes. In patients with cachexia, sarcopenia or frailty and HF, the progressive loss of muscle mass and strength is faster if there is also cancer in terminal stages, cirrhosis, or CKD; for these patients, maintaining muscle health and functional performance is of high importance [123,124,125]. Some studies indicate that the assessment of mass is less important than muscle strength [126]. Others suggest that muscle mass predicts adverse outcomes and mortality; however, the use of the techniques that assess muscle mass and/or commonly used body size normalizations have led to controversy [127].

Various bioelectrical and imaging techniques have been used to measure muscle mass such as muscle ultrasound, bioelectrical bioimpedance, dual-energy X-Ray absorptiometry, dilution deuterated creatine, single-slice computed tomography or magnetic resonance imaging [125,128,129]. These methods are used to assess body composition indirectly in HF. Bioelectrical impedance analysis is safe, fast, and easy to use and provides an estimate of body composition based on mathematical calculations. Nevertheless, it is expensive and needs to be validated in the cardiac cachexia. It also has limited use in patients with hydration alterations [129,130].

Patients that have an elevated risk of death may be identified by quantification from axial MRI images of the size of the pectoralis major muscle of the chest using a 1.5T whole-body MRI scanner. It is easy to obtain the pectoralis major area, and it is a reproducible measure [130,131]. Although the usefulness of this imaging method has gained importance, one of its limitations is the cost. In addition, there are few clinical trials using this method in cardiac evaluation and cachexia, although it could allow researchers to determine muscle status at the same time as the cardiac condition.

Since there is a close correlation between muscle mass of the entire body and the musculoskeletal state at the level of the L3 vertebra, an evaluation can be performed by computed tomography at this level. A single axial slice of the L3 vertebral body is selected, and Hounsfield Unit thresholds of −29 to +150 are applied to selectively identify skeletal muscle and exclude fat infiltration. The software calculates the cross-sectional area of skeletal muscle (cm^2^) by multiplying the pixel area by the number of pixels identified as skeletal muscle [132].

Regarding the follow-up of the nutritional status in patients with HF using biochemical parameters, albumin and prealbumin could be of use. In patients with advanced chronic HF and in patients undergoing placement of ventricular assistance devices, albumin is a useful indicator of adverse prognostic effects. Levels of albumin correlate with the level of fragility measured by low strength of the grip [133]. Since albumin is a negative acute phase reactant, its synthesis is inhibited in inflammatory conditions, and it should be combined with the determination of C-reactive protein [134]. In contrast, prealbumin has a diminished sensitivity to hydration and is considered a better marker of protein malnutrition. C-reactive protein should also be determined in combination with prealbumin since it is also a negative acute phase reactant, but, in contrast to albumin, it can be used to follow alterations in the same patient. Moreover, prealbumin levels correlate with mortality and higher readmission rates in subjects with acute HF [135,136].

Unintended loss of weight of 7.5% in patients with HF has been related to low total cholesterol, lymphopenia, anemia, and worse outcomes [22]. In addition, other modern biomarkers of nutritional status, such as ghrelin, leptin, adiponectin, myostatin or growth differentiation factor 8 (GDF-8) and the C-terminal fragment of agrin have been considered as possible biochemical parameters. Regarding ghrelin, its levels are elevated in patients with HF and cardiac cachexia compared to those with only HF [136]. Leptin release is inhibited in HF patients with cachexia when compared to noncachectic subjects. Adiponectin is elevated in patients with cardiac cachexia and is positively correlated with weight loss and brain natriuretic peptide [22]. There is an elevated risk of mortality in HF patients with elevated levels of adiponectin [137]. GDF-8 is a protein that is found mainly in skeletal muscle and is characterized by inhibiting its growth. It seems to be upregulated in animal models with volume overload in HF [138,139].

In contrast, the force of apprehension, measured by dynamometry, has been used as an isolated surrogate marker of muscular strength and frailty [117], and the reduction in force is associated to a higher rate of postoperatory complication and an increase in mortality in patients with advanced HF in whom a DAVI was implanted [117].

## 7. Treatment of Cachexia

At present, there is still no cure for cachexia or cardiac cachexia. In contrast to anorexia and malnutrition, which can be corrected with an adequate nutrition, cardiac cachexia is a significant challenge for treatment and prevention, and this is discussed in the next sections. In addition, nonpharmacological measures aimed to maintain aerobic physical activity have been proposed and are discussed [140]. Nevertheless, nutritional interventions, exercise, Vitamin D, and several drugs have been proposed to benefit patients.

### 7.1. Nutritional Interventions

Cachexia is related to malnutrition and all cachexia except possibly cancer cachexia, responds to judicious nutritional support. However, it is more responsive of nutritional therapy than malnutrition and sarcopenia. Cachexia must be treated cautiously to avoid overfeeding syndrome, which may result in serious or dangerous complications or death [111]. For a long time, the therapeutical approach for treatment of HF with or without cachexia had been centered only on the myocardium and the cardiovascular system. However, since it is often related to cachexia, other approaches aimed at metabolism and nutrition are currently being employed and tested as possible new successful therapies [141]. However, there are still no integral guidelines on dietary advice for this combination of diseases nor large clinical trials for patients with HF that could help correct cachexia or sarcopenia. Simply increasing protein and calorie intake, for example, by adopting the PROT-AGE Protein Targets (a target protein intake of 1.0 to 1.2 g/kg body weight/day) could possibly be of help [142]. In individuals with cardiac cachexia independently of its association with HF, it is recommended to begin feeding with a low kcal/kg ratio and increase the administration of the nutrient supply slowly and progressively avoiding large volumes with low energy density [143]. When patients with cardiovascular disease need nutritional support, supplementation either oral or enteral is the first option. In addition, HF is frequently associated with alterations in the morphology of the gut, permeability, and absorption, which complicate the administration of nutritional support [19]. Oral nutritional supplementation is indicated when oral feeding is inadequate, and it may be hypercaloric and, if necessary, hyperproteic. These diets also improve the inflammatory state, quality of life and survival rate in subjects with HF [144,145]. Furthermore, no specific recommendations on enteral/parenteral nutritional routes for patients with HF are available. However, when the nutritional support is inadequate, the enteral administration of artificial nutrition is recommended, particularly when the gut is intact and available. Administration should be performed using a nasoenteric tube and, when microaspirations or gastric paresis are present, a distal position of the tube at the duodenal level is recommended [146].

### 7.2. Micronutrients

A high-quality diet includes micronutrients, such as vitamins and trace elements, which are necessary components of the diet participating as regulatory elements in cellular metabolism [147,148]. There are several studies that have described the possible role of micronutrients in patients with HF; however, there are no general recommendations in guidelines for clinical practice.

Treatment with Vitamin D stimulates appetite, normalizes the weight gain, improves skeletal muscle fiber size, muscle function, normalizes muscle collagen content and attenuates the muscle fat infiltration in cachexia resulting from CKD [149]. Vitamin D also has antihypertensive effects and improves endothelial function, and therefore, Vitamin D may reduce the antecedents of HF if it is timely administered [150,151]. Vitamin D also serves as an anti-inflammatory agent and suppresses serum concentrations of parathormone, which contributes to impaired heart function. Calcium supplementation may result in a slight improvement in hemodynamic variables, and therefore, it may be useful for the treatment of HF [152]. However, clinical trials using Vitamin D have shown contradictory results [153,154,155,156,157]. There are controversial results on the effect of other vitamins including vitamin E in HF in spite of its effect on the control of oxidative stress and reduction of free radicals [158,159]. There are also contradictory results with the use of vitamins B_1_, B_12_ and C, although at some doses vitamin C improved muscular force [160,161,162,163,164].

Coenzyme Q_10_ has also proven to have benefic effects [165,166,167]. Regarding iron, a decrease in fatigue was observed, and the risk of hospitalization due to HF was decreased [168,169,170].

### 7.3. Exercise

At present, the effect of nutritional support is enhanced with physical activity. Therefore, a combination of both is the main treatment option to counteract the consequences of sarcopenia and cachexia in HF [145,146]. Therefore, physical exercise should be considered as a complement to nutritional therapy. Cardiac rehabilitation programs in patients with HF improve functional capacity, exercise tolerance and quality of life. They also result in a reduction of hospitalization and mortality rates [171].

Exercise activates numerous pathways that potentiate protein synthesis and decrease degradation, thus diminishing muscle wasting and resulting in muscle growth. Exercise has anti-inflammatory and antioxidant effects and reduces myostatin signaling. Therefore, it abolishes protein degradation. IGF-1 levels are also increased with exercise to induce protein synthesis through activation of mTOR-suppressing FoxO signaling. Exercise also induces the expression of the transcription factor is PGC-1α, which downregulates proteolysis [12].

### 7.4. Treatment with Other Drugs

Definitive treatment options for management of cachexia and HF are still not available, and therefore, there are no therapies with a proven benefit and safety [172,173]. However, appetite stimulants, anabolic agents (including testosterone) in combination with the administration of nutritional supplements and anticatabolic treatments have been proposed. Some of these treatments have demonstrated efficacy in a few retrospective analyses and clinical trials. In others reports, their therapeutic potential is based only on a theoretical point of view. In addition, studies focused specifically on cardiac cachexia are lacking [173].

Appetite stimulants, essential amino acids, growth hormone, testosterone, electrical muscle stimulation, ghrelin and its analogues, ghrelin receptor agonists [174] and myostatin antibodies have been proposed for the treatment of cachexia [2]. The wasting stimulant blockers such as megestrol acetate, medroxyprogesterone acetate and cannabinoids have also been suggested.

Other drug classes of interest comprise angiotensin-converting enzyme inhibitors [175]; beta-blockers that increase weight, fat deposits and levels of leptin and decrease norepinephrine [176]; anabolic steroids [177]; beta-adrenergic agonists, even if they are known to have potentially harmful effects in these patients [178,179,180,181]; inflammatory substances; statins; thalidomide; proteasome inhibitors and pentoxifylline [182].

Since insulin resistance is one of the main mechanisms associated with the development of cachexia derived from cancer, and type-2 diabetes is the most frequent comorbidity in patients with cardiac cachexia, some drugs that increase insulin sensitivity such as metformin could be used as treatment for cachexia; however, more research is needed in this regard since results are still controversial [183,184].

## 8. Summary and Conclusions

Heart failure may lead to cardiac cachexia, which results in increased morbidity and mortality. Cardiac cachexia is the loss of muscle mass, while fat tissue may decrease or remain stable. Loss of muscle mass is caused by an imbalance between protein synthesis and degradation, or it may result from intestinal malabsorption. The loss of balance in protein synthesis and degradation may be the consequence of altered endocrine mediators such as insulin, insulin-like growth factor 1, leptin, ghrelin, melanocortin, growth hormone and neuropeptide Y. Fat accumulation in the heart, which may be epicardial, myocardial or cardiac steatosis, is protective in this condition. Possible screening proposals are beginning to be tested in clinical trials, and the results will be important to allow for a timely detection of this condition since early identification and treatment is associated with better outcomes, and at present, there is no cure for this condition. The lack of a proven tool to assess the nutritional status in patients with HF renders it difficult to design an adequate intervention and has yet to be established. Muscle ultrasound seems to be an appropriate measurement technique, taking into account its low cost, availability, simplicity and good correlation with magnetic resonance imaging. Moreover, definitions of cutoff points for low muscle mass and fat tissue are still not available. There is no cure for cardiac cachexia, and several possible therapeutic measures have been proposed, among which nutritional intervention and exercise are included. Nevertheless, large randomized and controlled trials to test the usefulness of nutritional interventions are necessary. Physical activity as a complement to nutritional therapy has a great potential, but efforts are needed to determine the intensity, duration, periodicity, and type of exercise that would help most. Regarding refeeding, it is difficult to establish the correct estimation of its incidence and limits, and well-designed studies that would allow for general results. Pharmacological interventions proposed include myostatin antibodies, ghrelin, megestrol acetate, medroxyprogesterone acetate, cannabinoids anabolic steroids, beta-adrenergic agonists, anti-inflammatory substances, statins, thalidomide, proteasome inhibitors and pentoxifylline. In conclusion, research regarding cardiac cachexia is still required to clarify mechanisms that underlie this condition and its interconnection with HF. Cardiac obesity plays a protective role of against it. Screening of the early phases of its development should be employed to provide for timely interventions since there is still no proven cure to treat patients with this condition.
